# Hydrothermally Treated Soybeans Can Enrich Maize Stiff Porridge (Africa’s Main Staple) without Negating Sensory Acceptability

**DOI:** 10.3390/foods8120650

**Published:** 2019-12-06

**Authors:** Martin Kalumbi, Limbikani Matumba, Beatrice Mtimuni, Agnes Mwangwela, Aggrey P. Gama

**Affiliations:** 1 Department of Human Nutrition and Health, Lilongwe University of Agriculture and Natural Resources (LUANAR), Box 219 Lilongwe, Malawi; martinkalumbi@yahoo.com (M.K.); bmtimuni@gmail.com (B.M.); 2 Food Technology and Nutrition Research Group—NRC, Lilongwe University of Agriculture and Natural Resources (LUANAR), Box 143 Lilongwe, Malawi; 3 Department Food Science, Lilongwe University of Agriculture and Natural Resources (LUANAR), Box 219 Lilongwe, Malawi; amwangwela@luanar.ac.mw (A.M.); aggreyg@yahoo.co.uk (A.P.G.)

**Keywords:** maize-based stiff porridge, enrichment, hydrothermally treated soybeans, acceptability

## Abstract

Maize-based stiff porridge, a starchy protein-deficient staple food, dominates among the populations in sub-Saharan Africa (SSA). Unfortunately, this is often consumed along with leafy vegetables since the majority of the population in this region lack resources for the purchase of high protein animal source foods, a situation that exacerbates protein-energy malnutrition. Considering this, the current study evaluated the effect of enriching maize-based stiff porridge with flour made from hydrothermally treated soybeans on consumer acceptability. A total of nine experimental flours were prepared from maize and maize-soybean mixtures following a 3^2^ factorial design involving two factors, namely maize flour type (whole maize, non-soaked dehulled maize, and soaked dehulled maize) and soybean flour proportion (0%, 20%, and 30%). A total of 125 adult consumers from a rural setting in Malawi evaluated maize-based stiff porridges made thereof using a 7-point hedonic scale. Subsequently, the participants were asked to guess an ingredient that was added to some of the test samples. The 10% and 20% soybean-enriched maize-based stiff porridges scored 5/7 and above, with some being statistically similar to plain maize-based stiff porridges. No participant recognized that soybeans were incorporated into the maize-based stiff porridges. The study has clearly demonstrated the potential of enriching maize-based stiff porridge with hydrothermally treated soybeans without compromising consumer acceptability. This innovation could significantly contribute towards reducing the burden of energy-protein under-nutrition in SSA.

## 1. Introduction

Protein-energy malnutrition is a major public health concern in sub-Saharan Africa (SSA) [1,2]. Beside food insufficiency, it is because diets in SSA are predominantly starchy with low levels of proteins. Maize (*Zea mays* L.), a cereal with low protein content (10%), is the key staple crop in SSA which is largely consumed as a main dish during lunch and dinner in the form of a stiff porridge. The stiff porridge is usually prepared using flour made from whole maize, non-fermented degermed-dehulled maize or fermented degermed-dehulled maize and is called *nsima* in *Malawi*, *nshima* in Zambia, *sadza* in Zimbabwe, *ugali* in Kenya and Tanzania, *mielie pap* in South Africa, and many other names across SSA. Unfortunately, the majority of the population in SSA lack resources for the purchase of high protein animal source foods such as meat, milk, eggs, and fish protein [3]. Consequently, they consume this protein-deficient staple along with vegetable relish, thus exacerbating protein malnutrition in the region [4].

Legumes are nutritionally valuable protein sources (20–45%) that offer a cheap option for upgrading the nutritive value of cereal-based foods. Owing to the high protein content, soybeans are the most popular legume used to enrich porridge served as breakfast for the whole family, and as a complementary food for infants and young children in SSA [5,6]. The soybeans are usually roasted or are extruded together with the maize to take care of anti-nutritional factors, off-beany flavor, and reduce the porridge preparation time. However, the roasted and extruded soybean products cannot be used to enrich stiff porridge due to the formation of flavor compounds. Consumers in sub-Saharan Africa are very accustomed to a maize-based stiff porridge that is essentially flat and flavorless, and any deviations from this norm are likely to be objectionable [7,8,9,10]. Likewise, it is also impractical to use raw maize-soybean flour mixture as it takes so long to cook and besides, the stiff porridge prepared in this manner retains objectionable off-beany flavors.

Off-beany flavors in soybeans described as ‘‘painty, bean, green, and unpleasant’’ are an activity of endogenous lipoxygenase enzymes which catalyze the insertion of oxygen into polyunsaturated fatty acids producing hydroperoxides, which subsequently participate in autoxidation process leading to the production of off-flavor compounds—particularly hexanal and heptanal [11]. Lipoxygenase works almost instantaneously during the grinding of soybeans in an ambient environment [12]. However, soymilk research has proven that it is possible to hydrothermally deactivate lipoxygenase before it catalyzes the oxidation of polyunsaturated fatty acids at 80 °C or higher and produce acceptable milk [12,13,14]. 

Despite the feasibility and widespread application of hydrothermal deactivation of lipoxygenase in soymilk industry, no report is available about the evaluation of this technique in the preparation of odorless products for the enrichment of protein-deficient maize stiff porridge. Therefore, the objective of this study was to investigate the effect enriching maize-based stiff porridge with flour made from hydrothermally treated soybeans on consumer acceptability. In this study, the effect of soybean proportion and the maize flour type are investigated, and the nutrition implication of soybean enrichment is discussed. 

## 2. Materials and Methods

### 2.1. Experimental Flours 

**Figure 1:**
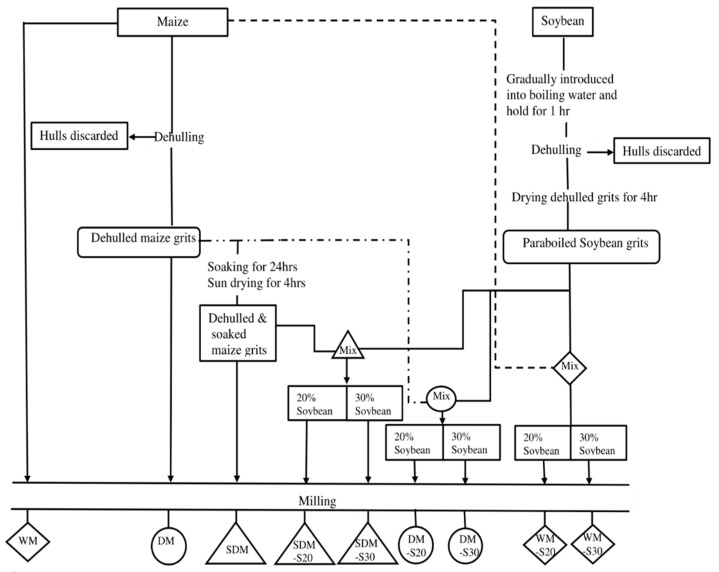
Scheme of flour preparation. DM, dehulled maize; DM-S20, dehulled maize with 20% soybean; DM-S30, dehulled maize with 30% soybean; WM, whole maize; WM-S20, whole maize with 20% soybean; WM-S320, whole maize with 30% soybean; SDM, soaked dehulled maize; SDM, soaked dehulled maize; SDM-S20, soaked dehulled maize with 20% soybean; SDM-S30, soaked dehulled maize with 30% soybean.

A total of nine experimental flours were prepared from maize and maize-soybean mixtures following a 3^2^ factorial design involving two factors, namely maize flour type (whole maize, non-soaked dehulled maize, and soaked dehulled maize) and soybean proportion (0%, 20%, and 30%) as summarized in Figure 1. 

The soybeans (Tikolore variety, obtained from Chitedze Research Station, Lilongwe, Malawi) were first hydrothermally treated to take care of the beany-grassy flavor in the following manner: Clean soybeans were gradually introduced into boiling water without causing the water to stop boiling (one part soybean to three parts water) for an hour, cooled in water, and manually dehulled by means of scrubbing the soybeans between two hands to force the hulls from the cotyledons. The dehulled soybeans were then washed and sundried for two days. To ensure homogeneity of the maize-soybean flour mixtures, the dried thermally processed soybean beans were thoroughly mixed with the maize portion (dry maize or dry dehulled maize grits) wherever applicable before the milling process (see Figure 1). In this study the authors assumed general nutrient composition data available in literature for soybean and a maize. In general, soybeans contain approximately 40% proteins and 20 % lipids [15], while maize contains only about 10% proteins and 4.5% lipids [16]. 

### 2.2. Preparation of Maize Stiff Porridges and Warming 

Five experienced women were engaged to jointly prepare nine maize stiff porridges, using each of the nine flours described in Figure 1, 2–3 h before the sensory evaluation test. The maize stiff porridge recipe involved cooking a mixture of 1500 mL of water and 600 g of grain flour for 40 min on an electric cooker, which was then immediately transferred into in tightly fitted food warmer ready for sensory evaluation. 

### 2.3. Consumer Acceptability

A total of 125 adult consumers (aged 18 years and above), without soy allergy, who regularly consume maize-based stiff porridge, were recruited from Lilongwe, rural Malawi, for this study after giving their informed consent. The recruits (participants) were predominantly female (63.2%), and more information about the participants is given in Supplementary Table S1.

Considering that consumer opinions are influenced by how much information has been presented to them about the products before tasting [17,18], the panelist were not informed that some of the test samples (maize-based stiff porridges) had been enriched with soybean flour. However, to screen out soy allergic consumers without compromising the blinding of participants (regarding the presence of soybean in some test maize-based stiff porridges), the participants were individually asked to indicate if they have problems with common allergens including fish, pork, meat, egg, peanut, tree nut, wheat, and soy. 

Each participant independently evaluated all of the nine maize-based stiff porridge samples (~8 g of each sample). The samples were presented one after the other, coded with random 4-digit numbers. The samples were served on white plates using a completely randomized balanced block design. In additional to asking the participants to rinse their palates with distilled water, five-minute breaks were also included between sample evaluations to minimize carry-over effects. A 7-point facial hedonic scale (1 = super bad, 7 = super good) was used to score the participant’s overall acceptability of each sample to accommodate those who did not attend any form of education or had low-level education attainment (Supplementary Table S1). A score of 5 (good) was taken as the lower limit of acceptability.

Upon evaluating the nine test samples, the participants were asked to guess an ingredient that was added to some of the test samples. Finally, the participants were informed about the inclusion of soybean and the processes involved. 

### 2.4. Data Analysis

A two-way ANOVA was used to test the main effects and interactions among the independent variables (maize flour type and soybean proportion). Post-hoc mean separations were carried out using Tukey’s honestly significant difference test at a significance level of 0.05 (*p* < 0.05). All statistical analyses were done in XLSTAT 2017 (version 19.01, Addinsoft, New York, NY, USA).

## 3. Results

**Figure 2:**
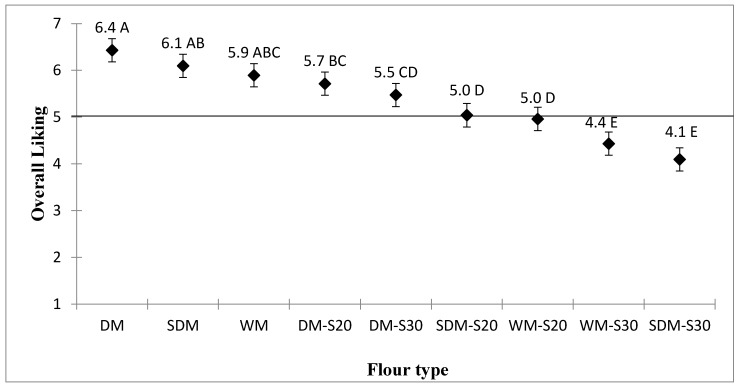
Overall liking mean score of maize-based stiff porridges. DM, dehulled maize; DM-S20, dehulled maize with 20% soybean; DM-S30, dehulled maize with 30% soybean; WM, whole maize; WM-S20, whole maize with 20% soybean; WM-S20, whole maize with 30% soybean; SDM, soaked dehulled maize; SDM-S20, soaked dehulled maize with 20% soybean; SDM-S30, soaked dehulled maize with 30% soybean. The different letters indicate significant differences among the flour types.

Results of overall hedonic ratings of the maize-based stiff porridge samples are presented in Figure 2. Irrespective of maize flour type, all blends incorporating 20% soybean (DM-S20, SDM-S20, and WM-S20) were regarded as acceptable with average hedonic scores of at least 5, the lower limit for acceptability on the 7-point hedonic scale. Except for the maize-based stiff porridge prepared from flour blend of 30% soybean and dehulled maize flour (DM-S30), incorporation of 30% soybean had a negative effect on acceptability. 

**Figure 3:**
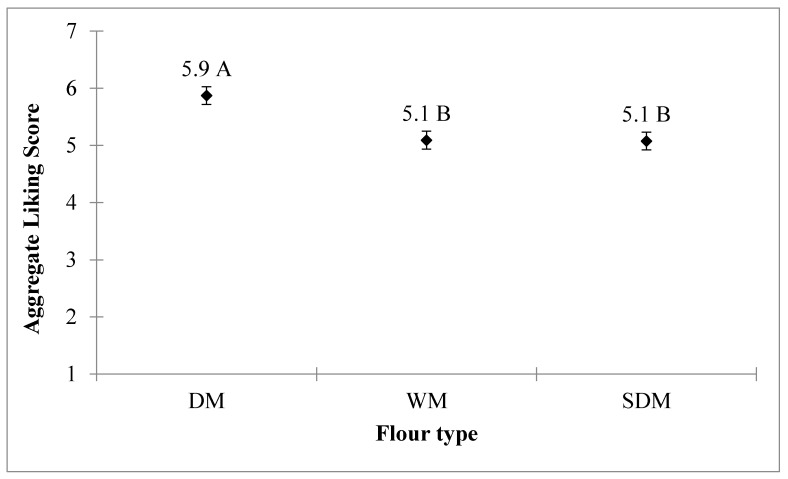
Effect of flour type on acceptance of the resultant stiff porridges. The aggregated liking score is the mean score computed based on flour type, irrespective of the proportion of soybean in the flour mixture. DM, dehulled maize; WM, whole maize; SDM, soaked dehulled maize. The different letters indicate significant differences among the flour types.

However, stiff porridges made from blended flours of non-soaked dehulled maize (DM), irrespective of soybean proportion, had relatively higher acceptability scores that were not significantly different from soaked dehulled flour (SDM) and whole maize (WM) plain flours, respectively (*p* ˃ 0.05). It is therefore not surprising that maize flour type, soybean proportion, and the interaction between maize flour type and soybean proportion had a significant effect on the acceptability of the resultant stiff porridges as shown in Table 1 as well as Figure 2 and Figure 3. 

When asked to guess what was added to the stiff porridge, none of the participants successfully recognized that soybean flour was incorporated in the maize-based stiff porridges. Nonetheless, participants reported that they suspected that something might have been added to some of the maize-based stiff porridges and distorted their texture. 

## 4. Discussion 

Owing to the high prevalence rates of malnutrition in SSA, a search for practical ways of improving nutrient densities of staple diets in the region is merited. This is the first study to report on the effect of incorporating of hydrothermally treated soybeans into maize-based stiff porridge on consumer acceptability. The results have shown that it is possible to enrich maize-based stiff porridge with a substantial proportion of hydrothermally treated soybeans without compromising consumer acceptability. Consumers in sub-Saharan Africa are very accustomed to a maize-based stiff porridge that is essentially flavorless, and any deviations from this norm is objectionable [7,8,9,10]. Therefore, the findings of this study suggest that unlike roasting, the hydrothermal treatment of soybeans did not result in the formation of other extraneous flavors, likely because of the unfavorable conditions for browning reactions such as Maillard reaction. 

Although most participants reported that they consume stiff porridge made from whole maize flour (WM) more often than from dehulled maize flour (DM) (Supplementary Table S1), in this study the participants highly liked stiff porridges made from DM. Likewise, among the blended flours, all blends of DM and soybean were highly ranked. Strange as it may seem, it is worth noting that sometimes socioeconomic restrictions override preferences, such that food is eaten just for survival in some situations [19,20]. The dehulling process results in approximately 20% loss of the maize flour quantity [21]. With the usual scarcity of food in SSA, people are likely to opt for WM even if they do not like it much to avoid the processing loss. It is, therefore, encouraging that WM blended with 20% soybean (WM-S20) was still acceptable as it could be a viable nutritious option even when food is scarce.

The novel maize-soybean stiff porridge provides a viable nutritious alternative staple for people living in the SSA. The stiff porridge made from maize-soybean blended flour not only contains higher amounts of protein, but also a higher content of lipids and micronutrients than the common maize-based stiff porridge. Since soybeans contain approximately 40% proteins and 20% lipids [15], while maize contains only about 10% proteins and 4.5% lipids [16], the novel 20% soybean stiff porridge typically represents a boost of 60% in protein and 69% in lipid composition (flour blend compositions: 16.0% protein and 7.6% lipid). This study preceded our published research, where we evaluated the efficacy of utilizing soybean-enriched stiff porridge as a strategy for managing human immunodeficiency virus (HIV)-related wasting among resource-poor people [22]. The 20% soybean-enriched stiff porridge induced a mean 2.9 kg cumulative mean weight gain among HIV-positive rural women within 3-month study period corresponding to body mass index (BMI) improvement from 19.3 to 21.1 kg/m^2^. 

It is worth highlighting here that there have been exploratory initiatives aimed at enriching staple foods with legumes before. Notably, Bressani et al. [23] evaluated the effect of enriching tortillas with soybeans. Later, Nyotu et al. [24] evaluated the effect of incorporating commercial defatted soy flour into stiff maize porridge using an untrained panel consisting of 12 Kenyan students, and recorded a positive outcome. Likewise, several researchers explored the enrichment of cassava-based stiff porridges (fufu) with cowpea and Bambara flours at a laboratory scale with success [25,26]. However, there is no record of the scaling up such innovations, possibly due to some socioeconomic hindrances. It is therefore imperative that future initiatives focus on understanding potential deterrents of the current innovation in order to make our innovation successful beyond the pilot scale. 

The study had certain limitations in that it did not systematically optimize the maize stiff porridges as it only investigated products with soybean proportions of 0, 20, and 30%. While stiff porridges containing 30% of soybean were less liked by the participants, it is possible that higher proportions of soybean between 20% and 30% would give a favorable outcome. Further, the study did not investigate the effect of various processes involved in the preparation of the soybean on nutrient retention and bioavailability. Such information would help to optimize nutrient losses and improve nutrient use efficiency. Nonetheless, given the positive outcome recorded in our research with HIV-positive women [22], there is clear evidence that the new soybean-enriched stiff porridge is superior to the traditional stiff porridge. 

## 5. Conclusions

The present study has demonstrated that it is possible to enrich maize-based stiff porridge with hydrothermally treated soybeans without compromising acceptability. Although the study involved participants from a single locality (Lilongwe, Malawi), the results herein may have wide application across SSA, particularly among the resource-limited poor who have minimal access to animal source protein foods, considering that soybeans potentially grow almost everywhere across the region. Unlike soybean-based ordinary porridges that are served as breakfast for the whole family and as complementary food for infants and young children, soybean-enriched maize stiff porridges have the potential of significantly contributing to the fight of energy-protein under-nutrition as stiff porridges are consumed by almost everyone in optimal quantities (dry matter basis) and regularly during lunch and supper. Future efforts should assess the feasibility of this innovation based on cost. 

## Figures and Tables

**Table 1:** Statistical results for effect of maize flour type and soybean proportion.

Variable	DF	Sum of Squares	Mean Squares	F	Pr > F
Maize flour type ^1^	2	154.483	77.241	38.496	<0.0001
Soybean proportion ^2^	2	418.332	209.166	104.246	<0.0001
Maize flour type * Soybean proportion	4	35.593	8.898	4.435	0.001

^1^ Maize flour types [dehulled maize (DM); whole maize (WM); soaked dehulled maize (SDM)]; ^2^ Soybean proportions (0% soybean; 20% soybean; 30% soybean); DF, degrees of freedom; F, Fisher-Snedecor distribution; Pr, probability. * stands for interactions between variables.

## Data Availability

The data that support the findings of this study are available from the corresponding author upon reasonable request.

## Supplementary Materials

The following are available online at https://www.mdpi.com/2304-8158/8/12/650/s1, Table S1: Demographic and socioeconomic characteristics of study participants and the type of the maize which they traditionally consume at their homes.
