# Supplementary material for: Hydrothermally Treated Soybeans Can Enrich Maize Stiff Porridge (Africa’s Main Staple) without Negating Sensory Acceptability
Source: Foods. 2019 Dec 6;8(12):650. doi: 10.3390/foods8120650 (PMC6963911; doi:10.3390/foods8120650)
Supplement: Supplementary file 1 [file foods-08-00650-s001.pdf]

Supplementary Table S1. Demographic and socioeconomic characteristics of study participants and the type of the maize which they traditionally consume at their homes.

	Gender, n (%) *		Age Group, n (%)							Education Level, n (%)		
	**Male**	**Female**	**18–20**	**21–30**	**31–40**	**41–50**	**51–60**	**61–70**	**>70**	**None**	**Primary**	**Secondary**
**Total**	46 (36.8)	79 (63.2)	16 (12.8)	45 (36.0)	26 (20.8)	19 (15.2)	11 (8.8)	4 (3.2)	4 (3.2)	16 (12.8)	74 (59.2)	35 (28.0)
**Flour type traditionally consumed by participants**												
**Soaked Dehulled Maize (SDM) flour**												
**Never**	24 (37.5)	40 (62.5)	10 (15.6)	23 (35.9)	12 (18.8)	6 (9.4)	7 (10.9)	3 (4.7)	3 (4.7)	8 (12.5)	34 (53.1)	22 (34.4)
**Rarely**	5 (35.7)	9 (64.3)	1 (7.1)	5 (35.7)	5 (35.7)	1 (7.1)	2 (7.1)	0 (0)	0 (0)	0 (0)	9 (64.3)	5 (35.7)
**Occasionally**	10 (43.5)	13 (56.5)	1 (4.3)	9 (39.1)	3 (13.0)	7 (30.4)	1 (4.3)	1 (4.3)	1 (4.3)	5 (21.7)	16 (69.6)	2 (8.7)
**Often**	7 (30.4)	16 (69.6)	4 (17.4)	7 (30.4)	6 (26.1)	5 (21.7)	1 (4.3)	0 (0)	0 (0)	3 (13.0)	14 (60.9)	6 (26.1)
**Always**	0 (0)	1 (100)	0 (0)	1 (100)	0 (0)	0 (0)	0 (0)	0 (0)	0 (0)	0 (0)	1 (100)	0 (0)
**Dehulled Maize (DM) flour**												
**Never**	19 (33.9)	37 (66.1)	1 (1.8)	20 (35.7)	15 (26.8)	11 (19.6)	4 (7.1)	3 (5.4)	2 (3.6)	11 (19.6)	35 (62.5)	10 (17.9)
**Rarely**	12 (46.2)	14 (53.8)	8 (30.8)	6 (23.1)	7 (26.9)	3 (11.5)	2 (7.7)	0 (0)	0 (0)	0 (0)	11 (42.3)	15 (57.7)
**Occasionally**	10 (41.7)	14 (58.3)	5 (20.8)	13 (54.2)	1 (4.2)	2(8.3)	3 (12.5)	0 (0)	0 (0)	2 (8.3)	16 (66.7)	6 (25.0)
**Often**	5 (31.2)	11 (68.8)	1 (6.2)	4 (25.0)	3 (18.8)	3 (18.8)	2 (12.5)	1 (6.2)	2 (12.5)	3 (18.8)	10 (62.5)	3 (18.8)
**Always**	0 (0)	3 (100)	1 (33.3)	2 (66.7)	0 (0)	0 (0)	0 (0)	0 (0)	0 (0)	0 (0)	2 (66.7)	1 (33.3)
**Whole Maize (WM) flour**												
**Never**	6 (30)	14 (70)	5 (25.0)	8 (40.0)	2 (10.0)	4 (20.0)	1 (5.0)	0 (0)	0 (0)	1 (5.0)	12 (60.0)	7 (35.0)
**Rarely**	2 (13.3)	13 (86.7)	0 (0)	4 (26.7)	7 (46.7)	2 (13.3)	1 (6.7)	0 (0)	1 (6.7)	3 (20.0)	9 (60.0)	3 (20.0)
**Occasionally**	5 (45.5)	6 (54.5)	1 (9.1)	4 (36.4)	1 (9.1)	2 (18.2)	1 (9.1)	1 (9.1)	1 (9.1)	2 (18.2)	9 (81.8)	0 (0)
**Often**	22 (44.9)	27 (55.1)	9 (18.4)	17 (34.7)	10 (20.4)	7 (14.3)	6 (12.2)	0 (0)	0 (0)	3 (6.1)	29 (59.2)	17 (34.7)
**Always**	11 (36.7)	19 (63.3)	1 (3.3)	12 (40.0)	6 (20.0)	4 (13.3)	2 (6.7)	3 (10.0)	2 (6.7)	7 (23.3)	15 (50.0)	8 (26.7)

* The values in bracket are percentages and those outside are frequencies as described in the heading of the table.
